# The impacts of different embolization techniques on splenic artery embolization for blunt splenic injury: a systematic review and meta-analysis

**DOI:** 10.1186/s40779-017-0125-6

**Published:** 2017-05-30

**Authors:** Jing-Jing Rong, Dan Liu, Ming Liang, Qing-Hua Wang, Jing-Yang Sun, Quan-Yu Zhang, Cheng-Fei Peng, Feng-Qi Xuan, Li-Jun Zhao, Xiao-Xiang Tian, Ya-Ling Han

**Affiliations:** 1 Department of Cardiology, General Hospital of Shenyang Military Region, Shenyang, 110016 China; 2 Department of Cardiology, Xinqiao Hospital of Third Military Medical University, Chongqing, 400038 China

**Keywords:** Blunt splenic injury, Embolization, Location, Material, Clinical outcome

## Abstract

**Background:**

Splenic artery embolization (SAE) has been an effective adjunct to the Non-operative management (NOM) for blunt splenic injury (BSI). However, the optimal embolization techniques are still inconclusive. To further understand the roles of different embolization locations and embolic materials in SAE, we conducted this system review and meta-analyses.

**Methods:**

Clinical studies related to SAE for adult patients were researched in electronic databases, included PubMed, Embase, ScienceDirect and Google Scholar Search (between October 1991 and March 2013), and relevant information was extracted. To eliminate the heterogeneity, a sensitivity analysis was conducted on two reduced study sets. Then, the pooled outcomes were compared and the quality assessments were performed using Newcastle-Ottawa Scale (NOS). The SAE success rate, incidences of life-threatening complications of different embolization techniques were compared by *χ* ^2^ test in 1st study set. Associations between different embolization techniques and clinical outcomes were evaluated by fixed-effects model in 2nd study set.

**Results:**

Twenty-three studies were included in 1st study set. And then, 13 of them were excluded, because lack of the necessary details of SAE. The remaining 10 studies comprised 2nd study set, and quality assessments were performed using NOS. In 1st set, the primary success rate is 90.1% and the incidence of life-threatening complications is 20.4%, though the cases which required surgical intervention are very few (6.4%). For different embolization locations, there was no obvious association between primary success rate and embolization location in both 1st and 2nd study sets (*P* > 0.05). But in 2nd study set, it indicated that proximal embolization reduced severe complications and complications needed surgical management. As for the embolic materials, the success rate between coil and gelfoam is not significant. However, coil is associated with a lower risk of life-threatening complications, as well as less complications requiring surgical management.

**Conclusions:**

Different embolization techniques affect the clinical outcomes of SAE. The proximal embolization is the best option due to the less life-threatening complications. For commonly embolic material, coil is superior to gelfoam for fewer severe complications and less further surgery management.

## Background

Splenic injury is the most common injury following blunt abdominal trauma in daily life and can lead to high mortality because of massive blood loss [1–5]. In the early 1990s, splenectomy was the only choice for splenic rupture. Because overwhelming postsplenectomy infection (OPSI) has occurred in 0.5% of all splenectomies in trauma patients and in over 20% of elective splenectomies [6], along with its associated high mortality (8–10%) and high postoperative infectious complications rate (45–49%), including pneumonia, bacteremia, urinary tract infections, abscesses, wound infections and so on [7–9]. So surgeons make every effort to preserve the spleen using various surgical and nonsurgical approaches [10]. Over the past few decades, nonoperative management (NOM) of blunt splenic injuries (BSI) in hemodynamically stable patients with or without splenic artery embolization (SAE) has been widely accepted and became the standard care currently [11–14]. There are growing evidences suggesting that SAE improves splenic salvage [15–19], as well as preserves the immunologic function of injured spleen [20–22]. However, the choices of different techniques (including embolization locations and materials) used in SAE were still at the interventional radiologists’ discretion or experience, and the superiority of different specific techniques is debated [23–28]. This situation may partly due to a vague scene of the effect and outcomes with different embolization techniques. Until now, no one prospective randomized study comparing the association between different embolization techniques with effects and outcomes of SAE is available. And in most published studies, the sample size of patients and the details of SAE were too limited to analysis and make a meaningful study. To improve the therapeutic efficacy and outcome, it is very necessary to further clarify the role of different embolization techniques of SAE.

## Methods

### Search strategy

Electronic databases of PubMed, Embase, ScienceDirect and Google Scholar Search were used to search published studies between October 1991 and March 2013 which related to the use of SAE as an adjunct to the NOM in patients of BSI. The Mesh terms used for search were “splenic” (“spleen”), “trauma” (“injury”), and “embolization” (“splenic artery embolization” or “SAE”).

### Inclusion criteria

Retrospective study evaluating BSI adult patients who underwent SAE were included and the following data were required: 1) basic demographics of patients; 2) indications for SAE; 3) the details of SAE techniques were given, including embolization locations (proximal, distal or combination) and/or embolic materials (coil or gelfoam); 4) the number of severe complications which were life-threatening or complications which need further surgical management.

### Exclusion criteria

The exclusion criteria were as follows: 1) single case report, reviews or editor letters; 2) studies involved only open injury, multiple trauma, pediatric patients or caused by iatrogenic injury; 3) non English language publications; 4) studies with insufficient or unconfirmed information.

### Study selection

The results of literature searching were screened preliminarily by two reviewers (ML and DL) using titles and abstracts. Then, the full texts of potentially appropriate literatures were searched for further screening.

### Data extraction

Data including the first author, publication year, number of patients, age, gender, indications for embolization, grades of American Association for the Surgery of Trauma-Organ Injury Scale (AAST-OIS), locations of embolization, embolic materials were extracted from studies, as well as the clinical outcomes including the number of successfully treated patients, severe complications which were life-threatening (rebleeding, infarction, abscess, cyst and contrast-induced renal insufficiency) and Dindo-Clavien classification of morbidity for complication III (DC III, see Table 1) which need further surgical management after SAE [7, 11, 15, 25, 26, 28–32].

**Table 1:** Clavien-Dindo classification of morbidity for complication

Grade I	Any deviation from the normal postoperative course without the need for pharmacological treatment or surgical, endoscopic and radiological interventions
	Allowed therapeutic regimens are: drugs as antiemetics, antipyretics, analgesics, diuretics and electrolytes and physiotherapy. This grade also includes wound infections opened at the bedside
Grade II	Requiring pharmacological treatment with drugs other than those allowed for grade I complications
	Blood transfusions and TPN1 are also included
Grade III	Requiring surgical, endoscopic or radiological intervention
IIIa	Intervention not under general anesthesia
IIIb	Intervention under general anesthesia
Grade IV	Life-threatening complication (including CNS complications) or requiring IC/ICU-management
IVa	Single organ dysfunction (including dialysis)
IVb	Multi-organ dysfunction
Grade V	Death

### Statistical analysis

The grades of AAST-OIS of patients among the included studies were compared using one-way ANOVA and then sensitivity analysis was performed on two reduced sets of studies to assess the impact on outcomes as a result of heterogeneity. First, all included studies were analyzed as 1st study set. Sequentially, studies were reanalyzed excluding those that lacked the necessary details of SAE (embolization locations, embolic materials and complications) and those without detail data of AAST-OIS grade or the outliers. The remaining studies were included in 2nd study set, and quality assessments were performed using Newcastle-Ottawa Scale (NOS). The total score over 6 was judged to be of good quality, otherwise, of poor quality.

SAE success was defined as spleen in situ after embolic treatment. The pooled rates of SAE success rate, incidences of life-threatening complications and DC III were compared among patients who treated by different embolization techniques by *χ* ^2^ test. Associations between different embolization techniques and clinical outcomes (including SAE success rate, incidences of severe complications and incidences of DC III) were evaluated by odds ratio (*OR*) and 95% confidence interval (CI) in 2nd study set, respectively.

Heterogeneity in 2nd study set was assessed using *Q* test, *P* and *I* ^2^ value. *P* > 0.05 for the *Q*-test indicated a lack of heterogeneity across studies, allowing to use the fixed-effects model (the Mantel-Haenszel method) [33–35]. The funnel plot and Begg’s test were used to examine the publication bias [36]. The *P* value was two-sided and of less than or equal to 0.05 was considered statistically significant. All statistical analyses were performed using Review Manager 5.0 and Stata 12.0 software. To ensure reliability and accuracy of the results, the data was analyzed by two researchers (JJR and QHW) independently and reached a consensus.

## Results

### Study selection and characteristics

**Fig. 1:**
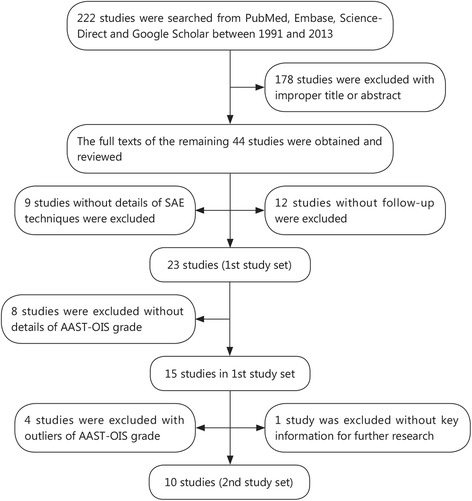
Flow diagram of study identification

Two hundred and twenty-two researches were found from literature search. After review of titles and abstracts, 44 studies were preliminarily identified for further evaluation. Of the 44 studies, 12 were excluded without follow-up [1, 12, 15, 17, 20–23, 37–40], and 9 studies were excluded because of the lack of detailed data about SAE techniques [19, 29, 31, 41–46]. Finally, 23 relevant studies [7, 10, 11, 16, 25–28, 30, 32, 47–59] were included as 1st study set. Then, 13 studies in 1st study set were excluded with significant differences of grades of AAST-OIS and the remaining 10 studies were included in 2nd study set (Flow diagram shown in Fig. 1). The NOS score of studies in 2nd study set was all over 6 and judged as good quality (Table 2).

**Table 2:** Quality assessment of studies in 2nd study set for meta-analyses using Newcastle-Ottawa Scale

Author	Year	Selection				Compatibility	Outcomes			Total
		1	2	3	4	5	6	7	8	
Liu et al.[10]	2004	▲	▲	▲	-	▲▲	▲	▲	▲	8
Franco et al.[25]	2011	▲	▲	▲	-	▲	▲	▲	▲	7
Wu et al. [26]	2011	▲	▲	▲	-	▲	▲	▲	▲	7
Smith et al.[27]	2006	▲	▲	▲	-	▲	▲	▲	▲	7
Haan et al.[28]	2004	▲	▲	▲	-	▲	▲	▲	▲	7
Ekeh et al.[30]	2005	▲	▲	▲	-	▲	▲	▲	▲	7
Wu et al.[32]	2008	▲	▲	▲	-	▲	▲	▲	▲	7
Killeen et al.[53]	2001	▲	▲	▲	-	▲	▲	▲	▲	7
Gaarder et al.[56]	2006	▲	▲	▲	-	▲▲	▲	▲	▲	8
Cooney et al.[57]	2005	▲	▲	▲	-	▲▲	▲	▲	▲	8

▲one point

▲▲two points

Overall, 876 patients who sustained blunt mechanism of injury and then received embolization treatment were included (Table 3). The average age in included studies was 38.8 ± 5.4 years (range 16–89) and most of the patients were male (72.4%, range 57.9–92.9%). The contrast blush (38.4%) or pseudoaneurysm (8.2%), high-grade (AAST III-V) splenic injury (50.1%), and large hemoperitoneum (18.0%) were widely considered as major indications for embolization after splenic injury [4, 7, 10, 11, 25–27, 30, 32, 47–51, 53–55, 57]. More than one indication per patient could occur and the distribution of these individual indications for embolization differed significantly in studies (Table 3). However, AAST-OIS is a quantitative and widely accepted grading scale for solid organ injuries which based on computed tomography (CT) image. The overall mean AAST-OIS grade of splenic injury was 3.4 ± 0.4 and differed significantly among studies in 1st study sets (range 2.9–4.5; *P* < 0.05, analysis of variance; Table 3). But within the 2nd study set, the differences were not significant (average 3.32 ± 0.20; range 3.1–3.7; *P* = 0.305, analysis of variance).

**Table 3:** Demographic characteristics of included studies

Author	*n*	Average age (year)	Male/Female	AAST	Indication [*n*(%)]			
					Contrast blush	Pseudoaneurysm	Large hemoperitoneum	AAST III-V
Niloy et al.[7]	45	48.0	28/17	3.0	27 (60.0)	7 (15.6)	0 (0)	31 (68.9)
Liu et al.^a^ [10]	6	43.8	4/2	3.7	2 (33.3)	0 (0)	3 (50.0)	6 (100.0)
Ekeh et al. [11]	88	37.8	59/29	3.4	17 (19.3)	21 (23.9)	0 (0)	79 (89.8)
Liu et al. [16]	15	-	-	3.4	-	-	-	-
Franco et al.^a^ [25]	14	44.8	13/1	3.1	8 (57.1)	-	-	6 (42.9)
Wu et al.^a^ [26]	53	37.5	33/20	3.3	33 (62.3)	11 (20.8)	0 (0)	-
Smith et al.^a^ [27]	41	-	-	3.1	-	-	-	27 (65.9)
Haan et al.^a^ [28]	140	33.0	106/34	3.5	107 (76.4)	0 (0)	9 (6.4)	87 (62.1)
Ekeh et al.^a^ [30]	15	36.0	11/4	3.5	8 (53.3)	-	-	14 (93.3)
Wu et al.^a^ [32]	19	46.5	11/8	3.5	8 (42.1)	1 (5.3)	10 (52.6)	19 (100.0)
Edmund et al. [47]	8	35.1	7/1	4.5	5 (62.5)	0 (0)	0 (0)	8 (100.0)
Ashraf et al [48]	109	-	-	-	-	-	-	-
Kaseje et al. [49]	11	32.7	-	-	11 (100.0)	0 (0)	0 (0)	-
Haan et al. [50]	32	-	-	-	0 (0)	32 (100.0)	0 (0)	-
Wu et al. [51]	10	-	-	-	8 (80.0)	0 (0)	2 (20.0)	-
Bessoud et al. [52]	37	40.0	28/9	3.7	14 (37.8)	0 (0)	34 (91.9)	-
Killeen et al.^a^ [53]	53	37.6	-	3.2	-	-	-	42 (79.2)
Sclafani et al. [54]	60	33.9	45/15	2.9	-	-	-	38 (63.3)
Sclafani et al. [55]	18	-	-	-	18 (100.0)	0 (0)	0 (0)	-
Gaarder et al.^a^ [56]	27	31.0	21/6	3.1	14 (51.9)	0 (0)	11 (40.7)	7 (25.9)
Cooney et al.^a^ [57]	9	39.0	6/3	3.2	6 (66.7)	0 (0)	0 (0)	9 (100.0)
Hagiwara et al. [58]	15	36.0	11/4	4.0	15 (100.0)	0 (0)	6 (40.0)	15 (100.0)
Wei et al. [59]	51	47.0	-	3.8	35 (68.6)	0 (0)	23 (45.1)	51 (100.0)
Total	876	38.8	634/242	3.4	336 (38.4)	72 (8.2)	158 (18.0)	439 (50.1)

^a^Study in 2nd study set

“-”not mentioned; AAST: Grades of American Association for the Surgery of Trauma-Organ Injury Scale

The overall primary success rate of SAE in 1st study set was 90.1% (range 72.7–100%; Table 4). Proximal embolization was performed more often than distal and combined significantly (52.1% *vs* 24.8% *vs* 5.5%). Exclusively proximal embolization, distal embolization and combination were performed in 5 studies [44, 48, 50, 52, 54], 1 study [10] and 10 studies [7, 11, 27, 28, 30, 47, 53, 55, 56, 58] (Table 4). On embolization materials, coil and gelfoam were commonly used materials in included studies. And only coil was used in 5 studies [11, 48, 50–52], gelfoam was exclusively used in 1 study [55].

**Table 4:** Details of the included studies

Author	*n*	SAE site (*n*)			SAE material (*n*)		Primary success [*n*(%)]	Severe complication [*n*(%)]	DC III [*n*(%)]
		P	D	P + D	Coil	Gelfoam			
Niloy et al. [7]	45	9	34	2	21	5	41 (91.1)	13 (28.9)	3 (6.7)
Liu et al. ^a^ [10]	6	0	6	0	5	1	5 (83.3)	0 (0)	1 (16.7)
Ekeh et al. [11]	88	51	22	15	88	0	84 (95.5)	8 (9.1)	0 (0)
Liu et al. [16]	15	-	-	-	0	0	12 (80.0)	1 (6.7)	0 (0)
Franco et al. ^a^[25]	14	8	6	0	12	1	13 (92.9)	1 (7.1)	0 (0)
Wu et al. ^a^[26]	53	5	48	0	24	29	44 (83.0)	14 (26.4)	0 (0)
Smith et al.^a^ [27]	41	27	9	5	22	2	30 (73.0)	12 (29.3)	12 (29.3)
Haan et al.^a^ [28]	140	-	-	-	83	48	122 (87.1)	38 (27.1)	3 (2.1)
Ekeh et al. ^a^[30]	15	10	1	4	-	-	14 (93.3)	1 (6.7)	0 (0)
Wu et al.^a^ [32]	19	3	16	0	0	16	14 (74.0)	7 (36.8)	5 (26.3)
Edmund et al. [47]	8	3	0	5	-	-	8 (100.0)	1 (12.5)	1 (12.5)
Ashraf et al. [48]	109	109	0	0	109	0	102 (93.6)	7 (6.4)	0 (0)
Kaseje et al. [49]	11	8	3	0	-	-	8 (72.7)	3 (27.3)	3 (27.3)
Haan et al. [50]	32	32	0	0	32	0	29 (90.1)	3 (9.4)	3 (9.4)
Wu et al. [51]	10	3	7	0	0	10	8 (80.0)	9 (90.0)	2 (20.0)
Bessoud et al. [52]	37	37	0	0	37	0	36 (97.0)	1 (2.7)	1 (2.7)
Killeen et al.^a^ [53]	53	24	22	7	-	-	47 (88.7)	43 (81.1)	3 (5.7)
Sclafani et al. [54]	60	60	0	0	60	0	57 (95.0)	5 (8.3)	3 (5.0)
Sclafani et al. [55]	18	17	0	1	18	0	18 (100)	1 (5.6)	0 (0)
Gaarder et al. ^a^[56]	27	21	2	4	-	-	26 (96)	1 (3.7)	0 (0)
Cooney et al.^a^ [57]	9	6	3	0	-	-	6 (67)	3 (33.3)	0 (0)
Hagiwara et al. [58]	15	9	1	5	-	-	15 (100)	5 (33.3)	1 (6.7)
Wei et al.[59]	51	14	37	0	-	-	50 (98)	2 (3.9)	15 (29.4)
Total	876	456	217	48	511	112	789 (90.1)	179 (20.4)	56 (6.4)

^a^Study in 2nd study set; SAE. Splenic artery embolization; P. Proximal splenic artery embolization; D. Distal splenic artery embolization; P + D: Combination of proximal and distal splenic artery embolization; DC III: Clavien-Dindo classification of morbidity for complication III; “-”Not mentioned

### Data synthesis of different embolization locations

#### Success rate

**Fig. 2:**
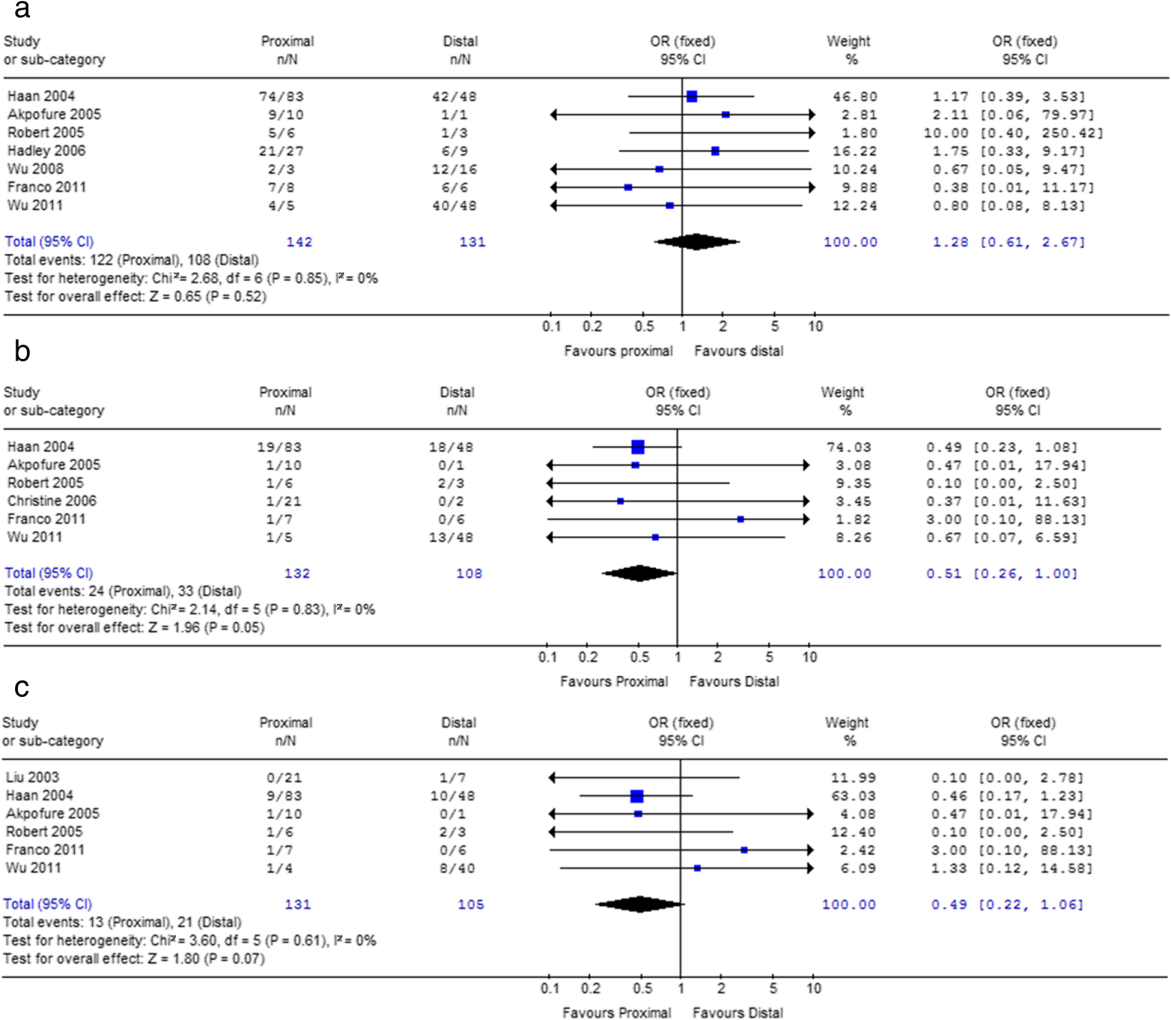
Forest plot of embolization locations (Proximal *vs* Distal) associated with success rate (**a**), severe complications (**b**) and the incidence of DC III (**c**) in 2nd study set

**Fig. 3:**
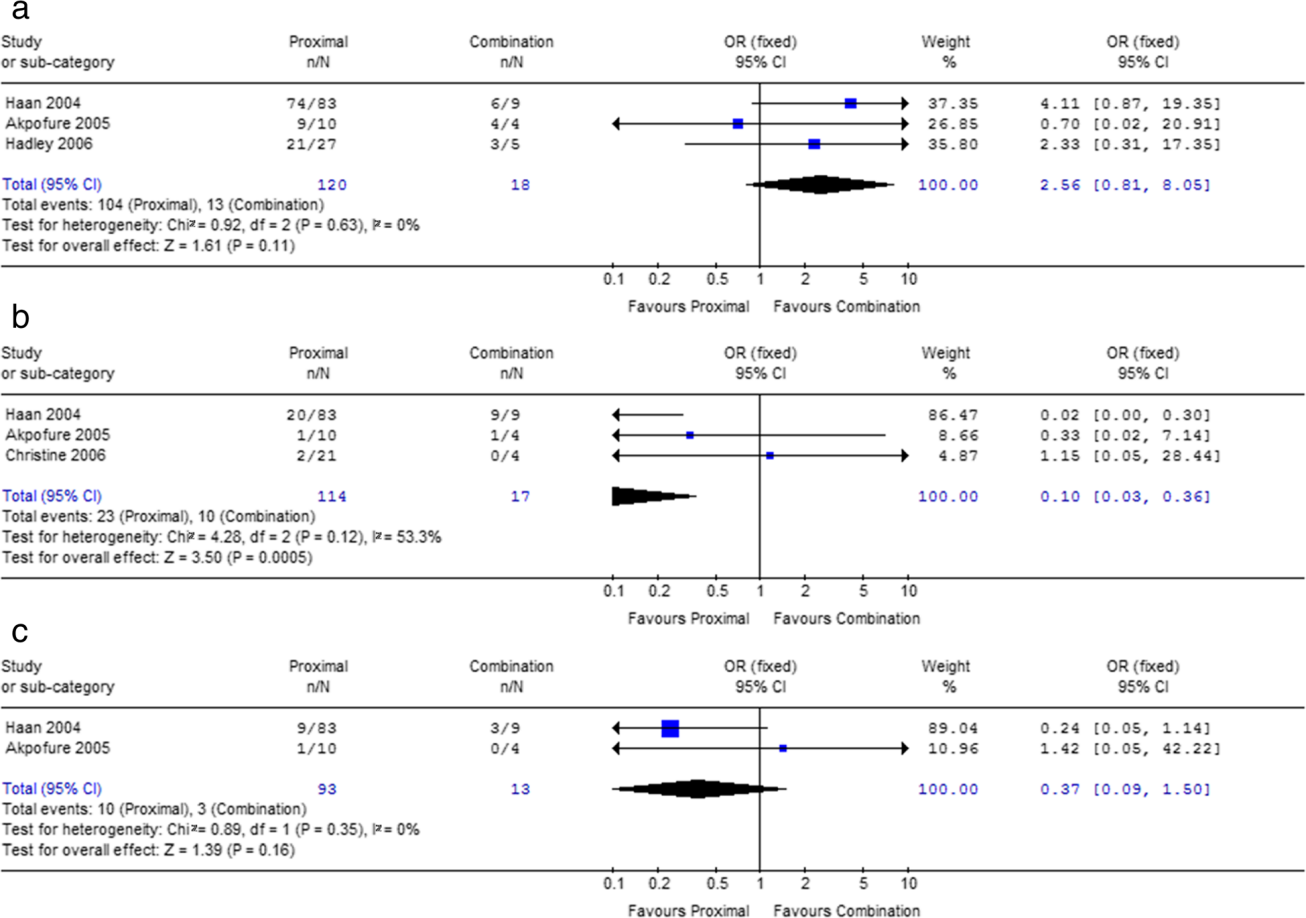
Forest plot of embolization locations (Proximal *vs* Combination) associated with success rate (**a**), severe complications (**b**) and the incidence of DC III (**c**) in 2nd study set

Due to the most common way, the proximal embolization (P) was compared with distal (D) and combination (P + D) on success rate. Within both 1st and 2nd study sets, the success rates of proximal embolization are all higher than distal embolization and combination (Table 5), but all these trends did not reach statistical significance (Figs. 2a and 3a).

**Table 5:** Comparisons of clinical outcomes between proximal *vs* distal and combined embolization in 1st and 2nd study sets

Outcome	Study set	Location	Percentage (%)	*P* value	*OR* (95% CI)
Success rate	1st	P *vs* D	91.4 (444/486) *vs* 87.7 (213/243)	0.11^a^	1.49 (0.91–2.45)
		P *vs* P + D	91.4 (444/486) *vs* 86.4 (38/44)	0.27^a^	1.67 (0.67–4.18)
	2nd	P *vs* D	85.0 (122/142) *vs* 82.3 (108/131)	0.52^a^	1.28 (0.61–2.67)
		P *vs* P + D	86.7 (104/120) vs 72.2 (13/18)	0.11^a^	2.56 (0.81–8.05)
Severe complication	1st	P *vs* D	10.7 (50/466) vs 30.7 (67/218)	<0.01^a^	0.27 (0.18–0.41)
		P *vs* P + D	10.7 (50/466) vs 35.6 (16/45)	<0.01^a^	0.22 (0.11–0.43)
	2nd	P *vs* D	18.2 (24/132) vs 28.7 (31/108)	0.05	0.51 (0.26–1.00)
		P *vs* P + D	20.2 (23/114) vs 58.8 (10/17)	0.00	0.10 (0.03–0.36)
DC III	1st	P *vs* D	7.3 (32/438) vs 13.0 (28/216)	0.02^a^	0.53 (0.31–0.90)
		P *vs* P + D	7.3 (32/438) vs 10.3 (4/39)	0.52^b^	0.69 (0.23–2.06)
	2nd	P *vs* D	9.9 (13/131) vs 20.0 (21/105)	0.07	0.49 (0.22–1.06)
		P *vs* P + D	10.8 (10/93) vs 23.1 (3/13)	0.16	0.37 (0.09–1.50)

P: Proximal splenic artery embolization; D: Distal splenic artery embolization; P + D: Combination of proximal and distal splenic artery embolization; DC III: Clavien-Dindo classification of morbidity for complication III

^a^Chi-square

^b^Fisher’s test

#### Incidence of the severe complications and the Clavien-Dindo classification III

In 1st study set, the average incidences of the severe complications, which were life-threatening, were 10.7, 30.7 and 35.6% for patients who underwent proximal, distal and combined embolization, respectively (Table 5). The differences between proximal and distal (*P* < 0.01), and between proximal and combination were all significant (*P* < 0.01). The results also showed higher incidences of complication DC III after distal embolization (13.0%) and combination (10.3%) than proximal embolization (7.3%). And there was significant difference between proximal and distal (*P* = 0.02, Table 5), however, the difference between proximal and combination was not significant (*P* = 0.52, Table 5).

In 2nd study set, the incidence of severe complications of proximal embolization was lower than distal (18.2% *vs* 28.7%) and combination (20.2% *vs* 58.8%, Table 5). Compared with proximal embolization, distal embolization increased the overall incidence of severe life-threatening complications [*P* = 0.05, *OR* = 0.51, 95%CI 0.26–1.00, *I* ^2^ = 0%, Fig. 2b], as well as combination embolization [*P* = 0.0005, *OR* = 0.10, 95%CI 0.03–0.36, *I* ^2^ = 53.3%, Fig. 3b]. The incidence of DC III of proximal embolization was lower than distal (9.9% *vs* 20.0%, Table 5) and combination (10.8% *vs* 23.1%, Table 5). Although the association was lost [Proximal *vs* Distal: *P* = 0.07, *OR* = 0.49, 95%CI 0.22–1.06, *I* ^2^ = 0%; Proximal *vs* Combination: *P* = 0.16, *OR* = 0.37, 95%CI 0.09–1.50, *I* ^2^ = 0%], there were trends that the more proximal embolization used, the less DC III occurred (Figs. 2c and 3c).

### Data synthesis of different embolic materials

#### Success rate

**Fig. 4:**
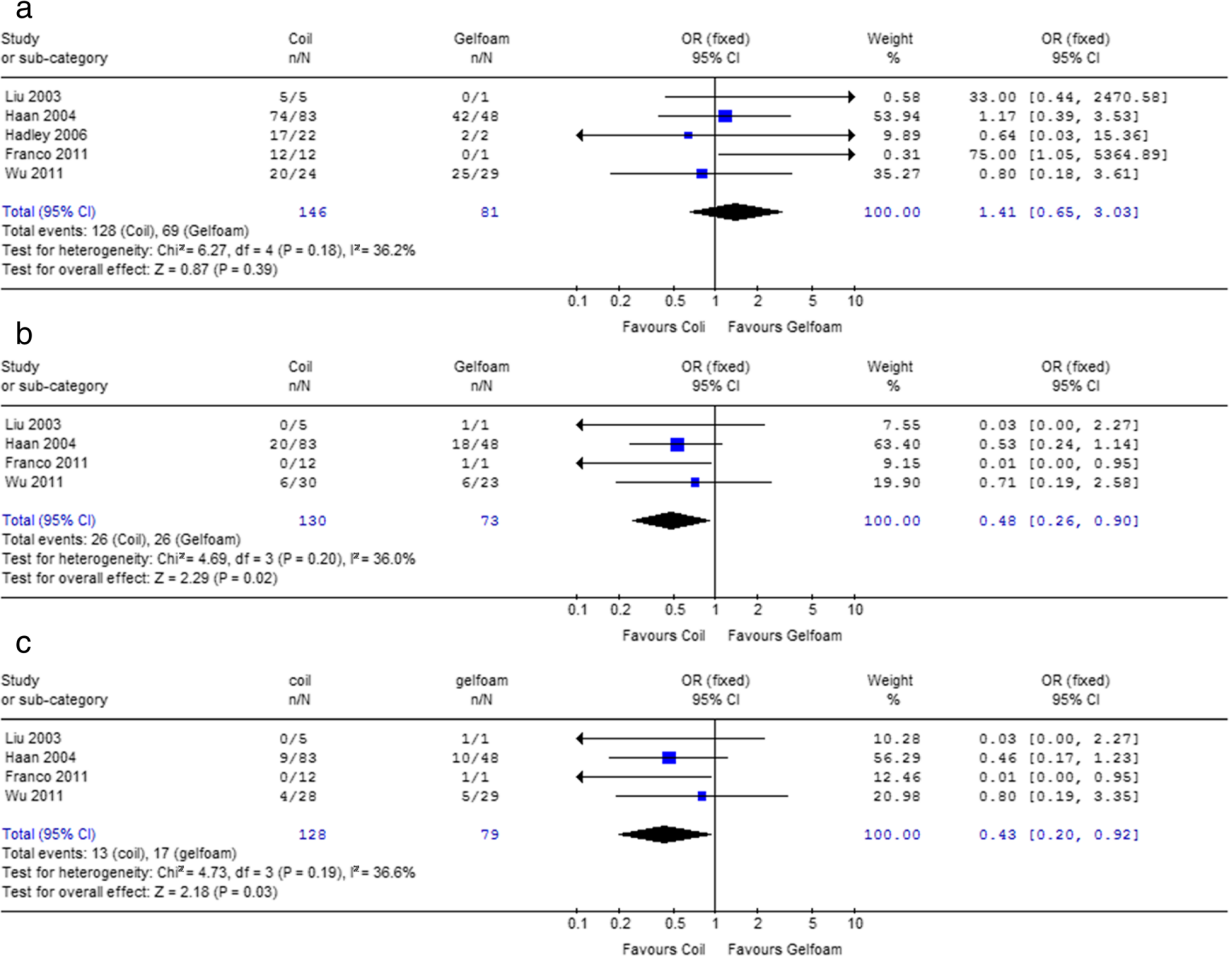
Forest plot of different embolic materials (Coil *vs* Gelfoam) associated with success rate (**a**), severe complications (**b**) and the incidence of DC III (**c**) in 2nd study set

As the most widely used materials, the therapeutic effects and clinical outcomes between coil and gelfoam were compared. The results of our study indicated that coil showed higher success rate than gelfoam (92.4% *vs* 83.9%) and the difference was significant (*P* = 0.006) in 1st study set (Table 6). However, within the five studies in 2nd study set, there was no association between embolic material and primary success rate [*P* = 0.39, *OR* = 1.41, 95%CI 0.65–3.03, *I* ^2^ = 36.2%, Fig. 4a].

**Table 6:** Comparisons of clinical outcomes between coil and gelfoam

Outcome	Study set	Coil (%)	Gelfoam (%)	*P* value	*OR* (95% CI)
Success rate	1st	92.4 (391/423)	83.9 (94/112)	0.0060^a^	2.34 (1.26–4.35)
	2nd	87.7 (128/146)	85.2 (69/81)	0.39	1.41 (0.66–3.03)
Severe complication	1st	12.5 (61/487)	41.6 (42/101)	<0.0001^a^	0.20 (0.12–0.32)
	2nd	20.0 (26/130)	34.2 (25/73)	0.02	0.48 (0.26–0.90)
DC III	1st	7.3 (36/493)	20.9 (23/110)	<0.0001^a^	0.30 (0.17–0.53)
	2nd	10.2 (13/128)	21.5 (17/79)	0.03	0.43 (0.20–0.92)

*DC III* Clavien-Dindo classification of morbidity for complication III

^a^Chi-square test

#### Incidence of the severe complications and the Clavien-Dindo classification III

In 1st study set, the average incidence of the life-threatening complications was significantly lower by using coil than gelfoam (12.5% *vs* 41.6%, *P* < 0.0001), as well as the incidence of DC III (7.3% *vs* 20.9%, *P* < 0.0001, Table 6).

Within the 2nd study set, strong associations were found between embolic material and life-threatening complication [*P* = 0.02, *OR* = 0.48, 95%CI 0.26–0.90, *I* ^2^ = 36%] or DC III [*P* = 0.03, *OR* = 0.43, 95%CI 0.20–0.92, *I* ^2^ = 36.6%]. The severe complication of coil was less than gelfoam significantly (20.0% *vs* 34.2%, *P* = 0.02, Fig. 4b). Our results also showed that the more the coil used, the lower incidence of DC III (coil *vs* gelfoam = 10.2% *vs* 21.5%, *P* = 0.03, Fig. 4c).

### Heterogeneity analysis and publication bias analysis

For both embolization locations and embolic materials study subgroups, there was no significant heterogeneity in each comparison (*P* > 0.05).

## Discussion

In our study, SAE represented an effective adjunct to NOM for adult patients of blunt splenic injury, and the therapeutic effect of SAE is prominent (primary success rate of SAE = 90.1%). The overall incidence of severe complications after SAE is 20.4%, though the cases which really required further surgical intervention is relatively fewer (incidence of DC III = 6.4%). For different embolization locations, there was no obvious association between primary success rate and embolization location in both 1st and 2nd study sets. However, our study suggests that proximal SAE reduced the risk of clinical adverse events than distal and combination, especially the life-threatening complications. As for the embolic materials, the using of coil is associated with a higher success rate and with a lower risk of developing life-threatening complication and DC III than using of gelfoam.

SAE may increase the nonoperative salvage rate in patients with splenic injury [10, 60, 61]. However, no clear consensus exists on patient selection criteria for SAE with regard to age or grade of spleen injury until now [1, 2, 37, 62–67]. So it is not surprising that recent practice management guidelines do not place much emphasis on SAE in managing BSI [62]. The use of abdominal CT scan has improved the ability to increase the accuracy of BSI diagnosis, to define the degree of injury, and to identify patients who are suitable for SAE [10, 19, 46]. Recent studies advocate good candidates for SAE in the presence of the following CT findings: contrast extravasation, pseudoaneurysm or arteriovenous fistula, large hemoperitoneum, and a high grade of injury (grade III–V) [4, 11, 19, 48, 59]. The AAST-OIS is a quantitative and widely accepted grading scale for solid organ injuries based on CT image. In order to further increase the reliability of our results, a sensitivity analysis was conducted on two reduced sets of included studies. By doing this, the differences in the grades of splenic injury should be eliminated in 2nd study set. And the main conclusions of this study are based on the analysis results of 2nd study set.

### Embolization locations

The choice of methods will have an effect on angioembolization efficacy and results [31, 42]. Embolization may be performed proximal (main splenic artery), distal (small arterial branches within the splenic parenchyma), or combination. Several researches over the past few years have reported conflicting clinical outcomes of different embolization locations [7, 10, 11, 23, 25–28, 32, 48, 53, 56, 57]. Proximal SAE achieves hemostasis by reducing pulse pressure and decreasing the distal flow of the splenic parenchyma. It is beneficial for clot formation rather than stop the hemorrhage directly [4, 23, 40, 47, 48], and it is advantageous to control multiple splenic injuries [23, 28]. Meanwhile, the rich network of collateral circulation from the left gastric, gastroepiploic arteries, pancreatic and omental branches enters the spleen to minimize the risk of infarction and preserve the function [12, 15, 20, 23, 25, 38, 39, 47, 48, 58]. In contrast, distal subselection embolization may not be feasible in these situations. Distal embolization occludes bleeding segmental vessels but may lead to wedge infarctions or increase the risk of abscess formation. And bleeding segmental vessels may be overlooked because of vasospasm caused by hematoma, potentially increasing the risk of rebleeding [7, 11, 28, 68]. In addition, proximal embolization is to simply occlude the main trunk of the splenic artery, which was approximately 2-3cm beyond the origin of the dorsal pancreatic and proximal to the first great pancreatic artery (arteria pancreatica magna) [15, 47, 54]. However, distal embolization is more technically challenging and has to occlude beyond the splenic hilum and distal to any major potential collateral pathways [15]. Therefore, proximal embolization is more technically simple and will be performed in less time and used less contrast agent. The reasons above could explain the results of our study that proximal SAE reduced the risk of life-threatening complications (including rebleeding, infarction, abscess and contrast nephropathy).

### Embolic materials

In the early 1980s, Sclafani et al. [40, 54] firstly introduced the concept of “angiographic hemostasis” using gelfoam, vasopressor and steel-wool coil injected into the splenic artery to treat blunt splenic trauma. Until now, coil and gelfoam still remain the most common agents of SAE. Coil is a kind of permanent and radio-opaque embolic agent [25], and can be injected from catheterization to the predetermined embolization location of injured splenic artery to block or decrease blood flow for clot formation and durable hemostasis. However, migration is a complication of coil often occurred during an embolization procedure and causes rebleeding or more infraction of spleen parenchyma [7, 11, 28, 30, 52]. Gelfoam is a kind of water-insoluble hemostatic agent prepared from purified skin gelatin. The first angiographic embolization used gelfoam was performed for hemostatic purpose prior splenectomy [29, 40]. Once injected, gelfoam follows the arterial blood stream and occludes the vessels, thus stopping the bleeding [25]. In addition, its hemostatic properties are also the result of entrapping the platelets in the porous and hastening the development and providing structural support to the thrombus [24]. One of the attractive characteristics of gelfoam is that it can be rapidly absorbed by macrophages and restoring vessel patency within days or a few weeks [25]. Instead, several papers report an increased risk of rebleeding before final hemostasis when gelfoam is used as embolic agent [10, 25, 27, 69, 70]. On the other side, once injected into the artery, the gelfoam particles would not only occlude the embolization location, but also occlude splenic vessels distal to collateral circulation with the flow, and splenic infarction was noted by ultrasound or CT follow-up [51, 54]. In addition, due to the capacity of retaining air bubbles that might give a chance for aerobic organisms to grow, the gelfoam eventually may lead to infection and abscess [24].

Our study indicated that the primary success rate of SAE was irrelevant to the embolic material. This result is consistent with Wu et al. [26] and Haan et al. [28]. At present, the association between embolization material and severe complication still remains controversial. It has been reported that the use of gelfoam resulted in the higher severe complication rate than coil [10, 25, 27, 32]. However, some other studies suggested that the difference was not significant [7, 26, 28]. According to our study, strong associations were found between different embolic materials and adverse clinical events. Coils should be the preferred embolic material used in SAE. Due to the higher incidence of life-threatening complications and DC III, an exclusively gelfoam embolization should be considered only as an urgent preoperative maneuver to deal with refractory shock and to enable patient transfer to the operating room [54].

## Conclusions

The studies in 2nd study set was judged as good quality (NOS > 6), and the results were reliable. This study demonstrated that the use of different embolization techniques would have significantly effects on clinical outcomes of SAE. Proximal embolization was the best option due to the less life-threatening complications than distal and combination. In addition, embolic material was another key factor which affected the outcomes of SAE. The use of coil in SAE surpassed using gelfoam, due to less adverse clinical outcomes. And more effective and reliable embolic materials would be further developed to improve the therapeutic effects of SAE.

## Abbreviations

AAST-OIS: American Association for the Surgery of Trauma-Organ Injury Scale
BSI: Blunt splenic injury
CI: Confidence interval
CT: Computed tomography
D: Distal embolization
DC III: Clavien-Dindo classification of morbidity for complication III
NOM: Non-operative management
NOS: Newcastle-Ottawa Scale
OPSI: Overwhelming postsplenectomy infection
OR: Odds ratio
P: Proximal embolization
P + D: Combination embolization
SAE: Splenic artery embolization
